# A Spatial Hierarchical Analysis of the Temporal Influences of the El Niño-Southern Oscillation and Weather on Dengue in Kalutara District, Sri Lanka

**DOI:** 10.3390/ijerph13111087

**Published:** 2016-11-04

**Authors:** Prasad Liyanage, Hasitha Tissera, Maquins Sewe, Mikkel Quam, Ananda Amarasinghe, Paba Palihawadana, Annelies Wilder-Smith, Valérie R. Louis, Yesim Tozan, Joacim Rocklöv

**Affiliations:** 1 Ministry of Health, Colombo 01000, Sri Lanka; dr_korelege@yahoo.co.uk (H.T.); ana_amarasinghe@yahoo.co.uk (A.A.); paba@health.gov.lk (P.P.); 2 Department of Public Health and Clinical Medicine, Epidemiology and Global Health, Umeå University, SE-901 87 Umeå, Sweden; sewemaquins@gmail.com (M.S.); mikkel.quam@gmail.com (M.Q.); annelies.wilder-smith@umu.se (A.W.-S.); joacim.rocklov@umu.se (J.R.); 3 KEMRI Centre for Global Health Research, Kisumu, Kenya, Box 1578, Kisumu 40100, Kenya; 4 Lee Kong Chian School of Medicine, Nanyang Technological University, Singapore 308232, Singapore; 5 Institute of Public Health, University of Heidelberg Medical School, D-69120 Heidelberg, Germany; valerie.louis@uni-heidelberg.de; 6 College of Global Public Health, New York University, New York, NY 10003, USA; tozan@nyu.edu

**Keywords:** dengue, vector control, Oceanic Niño Index, rainfall, temperature, weather, climate

## Abstract

Dengue is the major public health burden in Sri Lanka. Kalutara is one of the highly affected districts. Understanding the drivers of dengue is vital in controlling and preventing the disease spread. This study focuses on quantifying the influence of weather variability on dengue incidence over 10 Medical Officer of Health (MOH) divisions of Kalutara district. Weekly weather variables and data on dengue notifications, measured at 10 MOH divisions in Kalutara from 2009 to 2013, were retrieved and analysed. Distributed lag non-linear model and hierarchical-analysis was used to estimate division specific and overall relationships between weather and dengue. We incorporated lag times up to 12 weeks and evaluated models based on the Akaike Information Criterion. Consistent exposure-response patterns between different geographical locations were observed for rainfall, showing increasing relative risk of dengue with increasing rainfall from 50 mm per week. The strongest association with dengue risk centred around 6 to 10 weeks following rainfalls of more than 300 mm per week. With increasing temperature, the overall relative risk of dengue increased steadily starting from a lag of 4 weeks. We found similarly a strong link between the Oceanic Niño Index to weather patterns in the district in Sri Lanka and to dengue at a longer latency time confirming these relationships. Part of the influences of rainfall and temperature can be seen as mediator in the causal pathway of the Ocean Niño Index, which may allow a longer lead time for early warning signals. Our findings describe a strong association between weather, El Niño-Southern Oscillation and dengue in Sri Lanka.

## 1. Introduction

Dengue is an important, rapidly spreading, mosquito borne viral infection and is endemic in more than 100 tropical and subtropical countries around the world [1]. A recent study estimated that 390 million infections occur globally each year with 25% manifesting clinical symptoms, rendering dengue virus the most common mosquito born viral pathogen in humans [2]. Susceptible humans, dengue virus and *Aedes* mosquitos make up the cornerstones of the transmission cycle of dengue, but the transmission dynamics is further implicated by the environment, human behaviour, and globalisation [3,4].

Climate, as an environmental factor, plays a major role in global and local spared of dengue. Due to the location of Sri Lanka, within the tropics between 5°55′ and 9°51′ North latitude and between 79°42′ and 81°53′ East longitude, the climate of the island could be characterized as tropical. Rainfall in Sri Lanka has multiple origins. The dominant weather systems that are responsible for rainfall in Sri Lanka are the two monsoons and the convectional activity during the intervening periods. These four seasons are described as first inter-monsoon (FIM) from March to April, Southwest monsoon (SWM) from May to September, second inter-monsoon (SIM) from October to November and Northeast monsoon (NEM) from December to February. SWM and to a lesser extent NEM are the two most important seasons in terms of temporal distribution of rainfall in western parts of the country. Extreme phases of the El Niño Southern Oscillation (ENSO) with Oceanic Niño Index (ONI) more than 0.5 have been identified as a contributory factor on the seasonal rainfall of Sri Lanka [5,6,7]. Regional differences observed in air temperature over Sri Lanka are mainly due to altitude, rather than to latitude. The mean monthly temperature differs slightly depending on the seasonal movement of the sun, with some modified influence caused by rainfall. The mean annual temperature in Sri Lanka manifests largely homogeneous temperatures in the low lands and rapidly decreasing temperatures in the highlands [5].

Dengue is caused by a flavivirus with four distinct but closely related serotypes (DENV 1 to 4) and transmitted by female mosquito vectors of the *Aedes* species [8]. *Ae. aegypti* is the primary dengue vector in most endemic countries and a highly domesticated urban mosquito that lives close to human dwellings. It prefers to feeds on humans and lays eggs in artificial man-made containers. *Aedes albopictus* also contributes to dengue transmission, but serves as a vector primarily in rural areas [9].

The immature aquatic cycle of the dengue vector from egg to adult is about 7 to 9 days in conducive climate conditions [10]. The vector becomes infected by biting infected humans (or non-human primates) during the infective stage. Thereafter it can transmit the infection to non-infected humans following an extrinsic incubation period ranging from 5 to 12 days [11,12]. The clinical manifestations of dengue appear in the host following an intrinsic incubation period of 4 to 10 days. The host then goes through 2 to 10 days of viraemic or infective stage, completing the transmission cycle [8]. Infection can be asymptomatic; however, the disease leads to a wide spectrum of clinical symptoms and may result in clinical manifestations ranging from mild febrile illness to severe haemorrhagic fever and shock syndrome, irrespective of age and gender [13].

Dengue was first serologically confirmed in Sri Lanka in 1962. The first island-wide outbreak was reported in 1965 [14]. Progressively larger epidemics have occurred since early 2000s with inter-annual cyclic returning patterns, reaching much higher magnitudes after 2009 [15]. Dengue is now reported from almost all the districts in the country and considered to be hyper-endemic with co-circulation of all four serotypes. DENV-1 replaced DENV-3 in 2009 triggering a wave of severe dengue epidemic in Sri Lanka. During 2012 to 2014 all laboratory confirmed cases were due to either DENV-1 or DENV-4, with DENV-1 being the predominant serotype (85%) [16,17]. In 2012, 44,456 cases were reported corresponding to an incidence rate of 220 per 100,000 population [18,19,20]. Nearly 60% of the total dengue cases in the country are reported from Colombo, Gampaha and Kalutara districts in the Western province. Determinants of dengue epidemiology appear to vary island wide. In combination with weather variables, unplanned urbanization and lack of garbage disposal methods along with scarcity of recycling produce abundant breeding sites leading to the proliferation of disease vectors. The peak transmission period occurs usually in June following the SWM, followed by a less severe peak in December–January after the NEM in some districts [18,19].

Dengue control efforts in the country focus on disease and vector surveillance, integrated vector control, social mobilization for source reduction and emergency response activities during outbreaks in terms of intensified vector control measures and public awareness campaigns. A national-level multidisciplinary task force on dengue was established since May 2010 to coordinate the dengue control activities at national, provincial and district-levels. Each district has several Medical Officer of Health (MOH) divisions in which MOH is the responsible officer in implementing dengue control activities in the respective MOH division.

The effect of weather conditions on dengue vector life cycle and the vector’s ability to spread disease among humans (vectorial capacity) is well documented [12,21,22,23]. Rainfall affects vector abundance by replenishing breeding sites and stimulating egg hatching [24]. Even though heavy rainfalls may transiently reduce the risk of transmission by flushing larvae and pupae away from breeding sites or killing them, residual water collections increase the future risk for longer periods of time [25]. The risk of dengue transmission can increase with elevated temperatures by increasing the reproductive rate and biting rate of vectors and the probability of human to vector transmission per bite, as well as by reducing the extrinsic incubation period [12,21,26,27]. Other important factors affecting dengue epidemiology; urbanization, human behaviours, population growth and mobility, interactions between virus and hosts, and vector control programs [28,29].

Identification of time lags between the onset of certain weather observations and subsequent increases in dengue cases can provide important information on lead times for the implementation of response activities. Furthermore, the relationship of weather and climate to dengue incidence is important for an in-depth understanding of global climatic variability and change [30]. Scientific studies identifying and explaining such relationships in multiple areas by combining and contrasting temporally derived relationships between weather and dengue incidence at smaller geographical scales are, at present, lacking [31,32]. Understanding the heterogeneous spatio-temporal distribution of risk associations for dengue transmission is important in planning and implementing effective infection control measures [33]. In view of the increasing frequency and severity of dengue epidemics in Sri Lanka, it is important to understand how such associations can facilitate effective control of dengue in MOH divisions in a given district.

The objective of the study was to characterise the relationship and time lag between rainfall and temperature conditions and dengue incidence, to describe how the weather patterns associate to the ONI, and how the ONI associate directly to dengue incidence. We quantify a joint estimate of the relationships, and the spatial heterogeneity across MOH divisions of a district, using a statistical methodology developed for studying risks related to time varying exposures in multiple geographical areas. Kalutara district was selected because it is one of the highly dengue endemic districts in Sri Lanka and a wide variation is observed in geography and population densities.

## 2. Materials and Methods

### 2.1. Study Area

Kalutara district is situated adjoining to the Southern border of Colombo, the main metropolitan area of Sri Lanka. The geographical boundaries of the regions fall within the latitudes and longitudes of 6°47′ N and 6°91′ N and 79°57′ E and 80°18′ E respectively. It expands from the coastal region in the West to the edge of mountain ranges and rainforests in the central part of the island. Altitude is below 150 m in most parts of the district. The district has a population of around one million inhabitants over a land area of approximately 1501 km^2^ [34]. The average population density is 662 persons per km^2^ (ranging from 208 to 3352) [34,35]. Changes have been observed in land use (urban, semi-urban and rural) and the incidence of dengue across 10 MOH divisions the district. From 2009 onwards, the dengue incidence increased in Kalutara with increasing magnitude compared to previous era before 2009 [36]. Field entomological surveys conducted in MOH divisions in the district show that 50%–60% of *Aedes* breeding sites are domestic discarded receptacles found outdoors [36].

### 2.2. Data Collection

In Sri Lanka, an integrated surveillance system of communicable diseases includes dengue and has island-wide coverage through trained and dedicated clinical and public health staff. National surveillance data are based on timely, high-yield reports that capture symptomatic dengue patients classified according to a standard surveillance case definition based on 1997/2011 WHO classification [37,38]. Cases are notified to their respective MOH division of residence. Over the study period from 2009 to 2013, weekly dengue cases were extracted from this National Communicable Disease Surveillance System.

**Figure 1:**
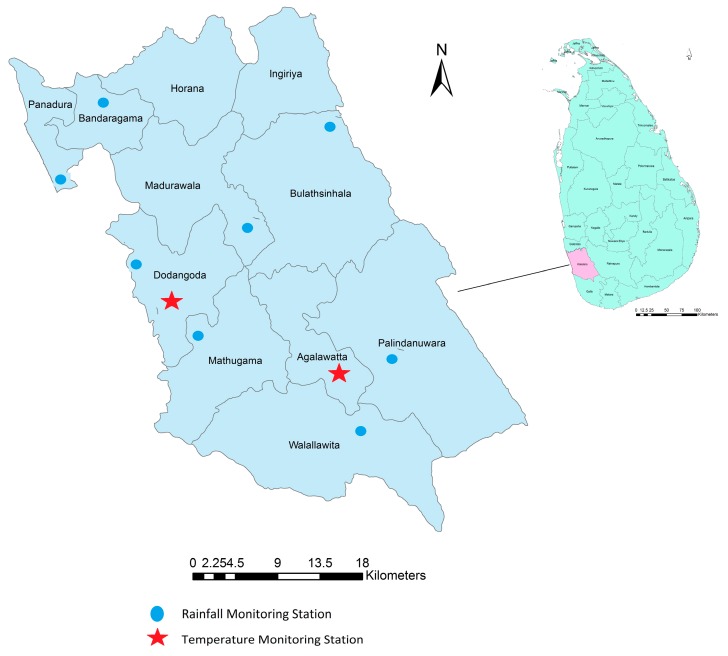
Map showing the MOH divisions and the geographical location of meteorological monitoring stations in Kalutara district, Sri Lanka. The solid line shows the boundaries of the MOH divisions in the district. The blue dots in the map represent the rainfall stations, and the red stars represent the temperature monitoring stations.

Daily rainfall and temperature data from 2009 to 2013 were extracted from eight rainfall and two temperature monitoring stations in the Kalutara district under the administrative purview of the Department of Meteorology, Sri Lanka. Figure 1 shows the geographical location of the weather monitoring stations and the MOH divisions in the district. Weekly cumulative rainfall and weekly mean temperatures were calculated using daily observations. The eight rainfall stations were located in eight different MOH divisions. For the remaining two divisions, the data from the nearest rainfall station were used. Over the study period there were a total of 102 days of missing rainfall observations distributed over different months in 6 of the monitoring stations with the longest missing duration of 28 days. The missing observations were imputed from the neighbouring area with the highest temporal correlation among the non-missing observations. There are, however, only two temperature monitoring stations in the district, and both showed a high temporal correlation with a coefficient of 0.85. Each MOH division was assigned the temperature recordings of the closest monitoring station. In addition remote sensing and gridded temperature data for all the MOH divisions were downloaded from FetchClimate [39] and Moderate Resolution Imaging Spectroradiometer (MODIS) [40] for validation purposes. ONI data as a measure of ENSO activity was obtained from NOAA Centre for Weather and Climate Prediction. The ONI tracks the running 3-month average sea surface temperatures in the east-central tropical Pacific between 120° and 170° W (Niño 3.4 region) [41].

The MOH division, Dodangoda, was created by dividing the MOH division, Matugama, into two divisions in 2011, but in the analysis these MOH divisions were combined to provide a longer time series of complete data for a total of 10 MOH divisions over the study period. We obtained permission to conduct the study in Kalutara district from the Ministry of Health through the provincial director of health services in Western province. No patient-specific information was collected for this study.

### 2.3. Statistical Analysis

A Poisson time series design with a two-stage hierarchical procedure was used for the analyses [42,43,44,45,46,47]. In the first stage, location-specific exposure-response relationships were flexibly estimated in relation to each MOH division, using Distributed Lag Non-linear Models (DLNM) [43]. In the second stage, estimates were combined using a non-linear multivariate meta-analysis model pooling the associations between weather variables and dengue incidence in all MOH divisions in the district [44].

#### 2.3.1. First Stage Division Specific Analysis

First-stage analysis was carried out in each MOH division establishing a generalized linear model with a quasi-Poisson distribution using the framework of DLNM [43,46]. During the model construction we included a flexible cross-basis function constituting splines for each variable and for the lag space. The cross-basis rainfall function was defined by a natural cubic spline function with 3 degrees of freedom (*df*) and by two internal knots placed at equally spaced values along rainfall (137 and 275 mm per week). The boundary knots are set to the average minimum and maximum rainfall values, ranging from 0 to 550.3 mm per week in each division. The reference value was set to 0 mm rainfall per week. The cross-basis function for temperature was defined by a natural cubic spline with one *df* for the space of temperature centred at 29.8 °C, which corresponds to the mean value of the temperature range. We placed one internal knot at 27 °C, and boundary knots according to the average minimum and maximum in each specific division at 25.6 °C and 33.6 °C. Based on existing literature and knowledge of biological processes in the vector in relation to human disease transmission [23,48,49], a lag term up to 12 weeks was included for weather variables. This decision was validated by examining cross-correlation coefficients of each variable and dengue cases. The lag dimension of the cross-basis functions was defined by a natural cubic spline function with a maximum lag of 12 weeks using 3 *df* for rainfall and 2 *df* for temperature. One internal knot was placed at lag of 6 weeks for rainfall and two internal knots at lags of 3 and 6 weeks for temperature. Two boundary knots were placed at 0 and 12 weeks for both variables. Further, to adjust for time trends caused by extraneous variables, such as shorter-term migration, population mobility and growth, we included a natural spline function of time with 1 *df* per year of observation [50]. We also accounted for changes in the size of the population by offsetting the mid-year population.

The general algebraic definition for the regression model is as follows:

Y(*t_i_*) ~ Poisson (µ(*t_i_*))

$$g\left(\mu_{ti}\right)=\beta_{i}+NS_{i}\left(rain_{ti},var\;df,\;lag\;df)+NS_{i}(temp_{ti},var\;df,\;lag\;df\right)+NS_{i}\left(trend_{ti},df\right)+\log\left(pop_{yi}\right)$$

where *g* is a log link function of the expectation *µ_ti_ ≡ E*(*Y_ti_*), with *Y_ti_* as the series of 260 weekly aggregates of dengue cases reported from 2009 to 2013 for each *i* MOH division. *t* represents the time in weeks from the start of the study. A location specific constant is estimated by β. The exposure variables *rain_ti_* and *temp_ti_* correspond to the flexible cross-basis functions of rainfall and temperature at week *t* over a lag dimension of 0–12 weeks estimated by degree of freedom of lag (*lag df*) and degree of freedom of variable (*var df*), as described above. The location specific natural cubic spline functions of rainfall, temperature and time trend are denoted by *NS_i_* and *pop_yi_* represents the yearly population in each *i* MOH division.

For the analysis of exposure lag response association between ONI-rainfall, ONI-temperature and ONI-dengue we used monthly mean rainfall, monthly mean temperature aggregated based on daily data and monthly cumulative dengue cases respectively. Three models were developed to describe each relationship using a similar approach described above.

The general algebraic definition for the model used for each response variable is as follows

Y(*t_i_*) ~ Poisson (µ(*t_i_*))

$$g\left(\mu_{ti}\right)=\beta_{i}+NS\left(ONI_{t},var\;df,\;lag\;df\right)+NS\left(trend_{t},df\right)+\log\left(pop_{yi}\right)$$

where Y(*t_i_*) represents a series of monthly mean rainfall, monthly mean temperature and monthly mean dengue cases over 60 months from 2009 to 2013 in each *i* MOH division respectively for each model. *t* represents the time in months. The exposure variable ONI corresponds to the flexible cross basis function for ONI at month *t* over lag dimension of 0 to 6 months. For the models ONI-rain and ONI-cases, this basis function was defined by naturel cubic b splines with one degree of freedom for the space of ONI (*var df*) and by three internal knots placed at ONI values −0.625, −0.2367 and at 1. The boundary knots are set to the minimum and maximum ONI values, ranging from −1.5 to 1.3. Only exception in the model ONI-temperature is the usage of natural cubic spline function for the ONI cross basis. The lag dimension of the cross-basis functions was common to all three models was defined by a natural cubic spline function with a maximum lag of 6 months using 2 *df (lag df).* Boundary knots were placed at 0 and 6 with an internal knot at 4 months. The location specific natural cubic spline function for ONI and time trend is denoted by *NS* and *pop_yi_* represents the yearly population in each *i* MOH division.

#### 2.3.2. Second Stage Meta-Analysis

The main aim of the second stage analysis is to derive a set of regression coefficients defining a representative exposure–response association generalized across the 10 MOH divisions for Kalutara district combined with parameters of heterogeneity. The study specific estimates obtained from the first-stage models are combined through multivariate non-linear meta-analysis. The associations are pooled together in a joint distribution using a restricted maximum likelihood approach. For the second stage meta-analysis, we estimated a pooled natural cubic spline function based on the set of divisional rainfall and temperature exposure response relationships. Heterogeneity in exposure-response associations among MOH divisions was assessed using the Cochran Q-test of heterogeneity, and was further quantified by the related *I*^2^ index [51]. The relative risk (RR) is calculated with reference to the risk at the centring reference values for temperature (29.7 °C) and rainfall (0 mm).

Different models were evaluated based on a modification of the Akaike information criterion (AIC) for quasi-likelihood models (Q-AIC) [52]. The selected model with the knot positioning described above had a lowest value of the sum of the Q-AIC in all 10 MOH divisions. The model consists of cross basis matrix of weekly cumulative rainfall, weekly mean of evening temperature and smooth function of time trend (in week). A description on the selection of the weather variables is given with the supplementary document (Table S1). The nature of the resulting exposure-response plots for pooled associations was also taken into consideration for model selection in terms of their capacity to describe biologically plausible associations. Further validation was done by plotting the predicted residuals against the observed data, considering the residual sequence plots at first-stage divisional analysis. Autocorrelation (ACF) and partial autocorrelation (PACF) were evaluated to assess uncontrolled dependence in the residuals to assure unbiased estimation of uncertainty associated with parameter estimates from each MOH division.

For all analytical estimations we used the *dlnm*, *mgcv* and *mvmeta* packages operated within the statistical environment of R [53]. A thorough methodological overview of DLNM and multivariate meta-analysis were described in detail in the published literature [43,44,45].

## 3. Results

A total of 7412 dengue cases were reported across all MOH divisions in Kalutara district from January 2009 to December 2013 with an average annual incidence rate of 151.8 per hundred thousand populations. Panadura MOH division, the most populated area situated close to the border with Colombo district, contributed to 40% of the total dengue cases reported in Kalutara district throughout the study period. Matugama, Horana and Bandaragama divisions routinely contributed to around 25% of the total dengue cases each year. Despite being a rural area with low population density, the Walallavita division reported a higher proportion of dengue cases during the 2012 dengue epidemic compared to other years. The rest of the divisions consistently reported low numbers of dengue cases annually during the study period.

Table 1 describes land area, population density, dengue incidence and weather patterns in all MOH divisions in Kalutara District, Sri Lanka, 2009–2013.

**Figure 2:**
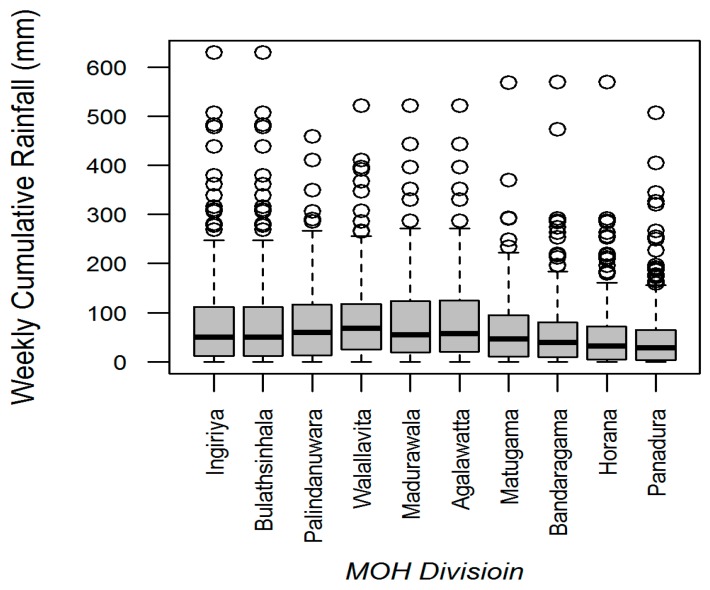
Distribution of weekly cumulative rainfall in all MOH divisions in Kalutara district, Sri Lanka, 2009–2013. The upper and lower borders of the box represents the interquartile range and the horizontal thick bar within the box indicate the median (50th percentile). The vertical lines extending from the boxes (whiskers) indicating variability outside the upper and lower quartiles. The circles above represent the outliers.

Figure 2 describes the distribution of rainfall in all MOH divisions during the study period in Kalutara district, Sri Lanka.

**Figure 3:**
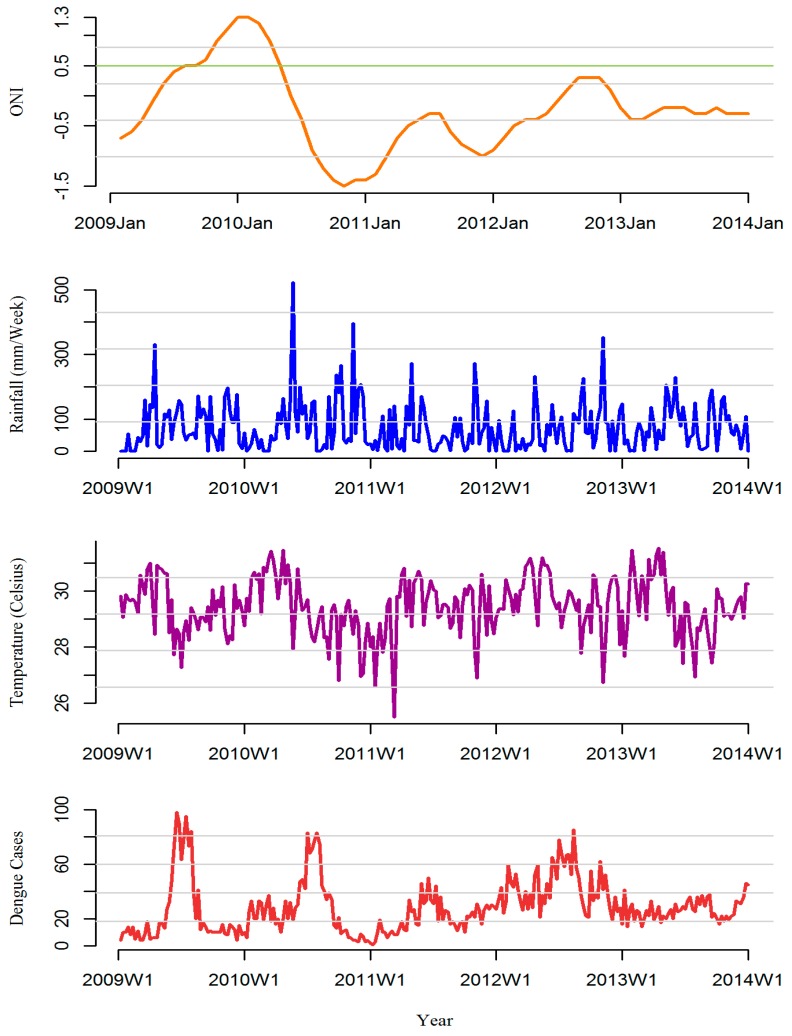
Time series of monthly ONI, weekly dengue cases, weekly cumulative rainfall and weekly mean temperature averaged across 10 MOH divisions in Kalutara district, Sri Lanka, 2009–2013. ONI: Minimum = −1.5, Maximum = 1.3; Total Dengue cases: Minimum = 1, Maximum = 98; Average Rainfall: Minimum=0 mL/week, Maximum = 527 mL/week; Weekly mean Temperature: Minimum = 25.5 °C, Maximum = 33.6 °C) Green horizontal line represents ONI of 0.5.

Figure 3 shows the monthly ONI, cumulative average of weekly weather predictors and dengue cases reported in all MOH divisions in Kalutara district, Sri Lanka, 2009–2013.

**Figure 4:**
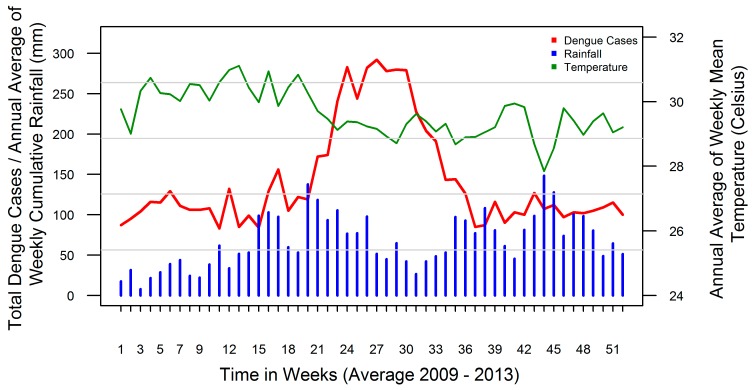
Week specific cumulative dengue cases, average rainfall and average temperature values recorded across all 10 MOH divisions in Kalutara district, Sri Lanka, 2009–2013.

Except for the period 2012–2013 during which a prolonged but flattened dengue epidemic was observed, all the other years displayed distinct annual seasonal peaks of dengue cases from May to September. The maximum number of cases per week for the study period was observed at the 24th week of the year 2009 with 98 cases. Since 2009, however, a gradual increasing trend for all weeks was noted. During the study period ENSO extreme event (ONI more than 0.5) reported only once from July 2009 to April 2010. The annual average rainfall in Kalutara was 3260 mm with the bulk of the rain falling during the SWM and NEM where highest rainfall reported during 2010. The lowest annual average rainfall of 1600 mm was reported in the coastal regions, and the highest, 4280 mm, was reported towards the inner mountain areas illustrating substantial heterogeneity among MOH divisions. Rainfall pattern showed bi-annual peaks coinciding with the monsoon periods in June following the SWM, and in November with NEM. Temperature was gradually increasing reaching its maximum around April and decreasing thereafter towards the end of the year (Figure 4). During 2012–2013, which was an epidemic period, there were more rainfalls throughout the district. The average temperature ranged between 25.5 °C and 33.6 °C [5]. The divisional dengue and weather variables followed the same annual pattern with some variation in the amount and the time scale.

### 3.1. Rainfall-Dengue Association

**Figure 5:**
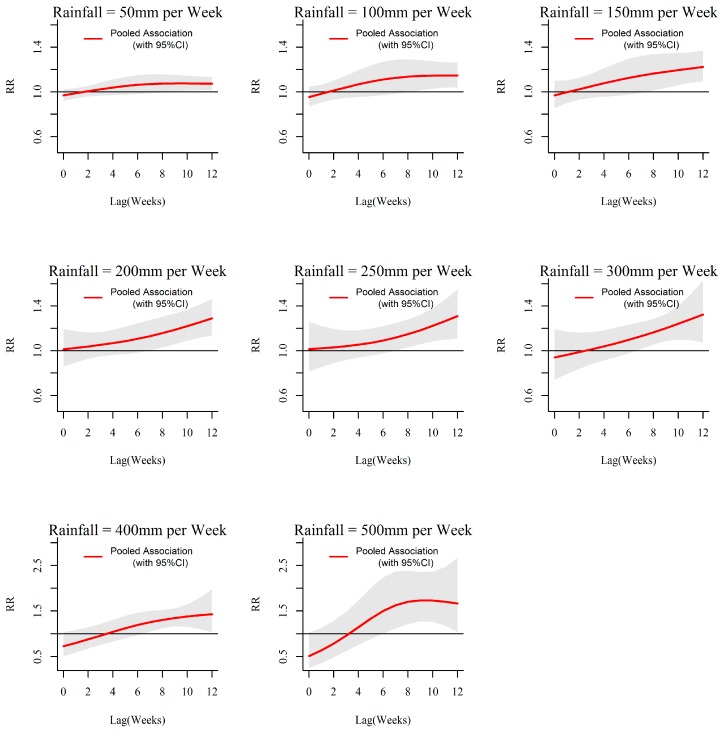
Relative risk (RR) by lag at specific cumulative rainfall levels predicted from the pooled exposure–response function for the 10 MOH divisions in Kalutara district, Sri Lanka, 2009–2013. The figure illustrates the predicted curves from meta-regression for different rainfall values of 50, 100, 150, 200, 250, 300, 400, and 500 mm per week. Shaded areas represent 95% confidence intervals. Reference at 0 mm per week.

**Figure 6:**
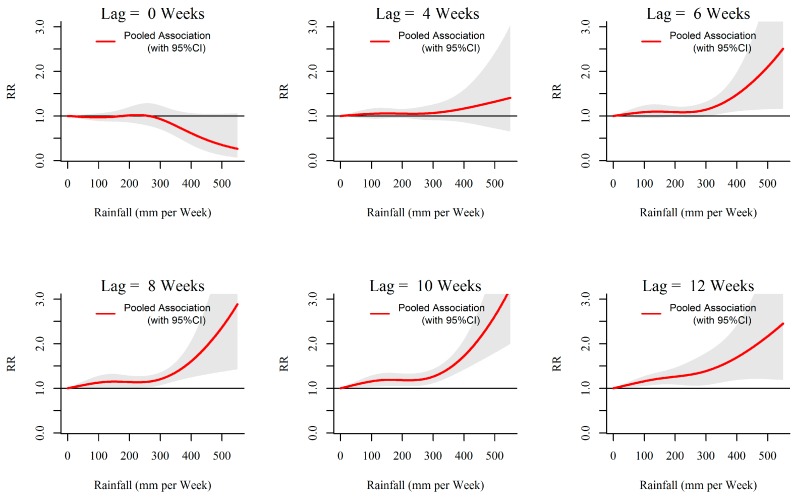
Relative risk (RR) of dengue by weekly cumulative rainfall at specific lags in 10 MOH divisions in Kalutara district, Sri Lanka, 2009–2013. The figure illustrates the predicted curves from meta-regression for different lag periods; 0, 4, 6, 8, 10 and 12 weeks. The shaded areas represent 95% confidence intervals. Reference at 0 mm per week.

The overall rainfall-dengue associations in the 10 MOH divisions in Kalutara district obtained through meta-analysis are illustrated in Figure 5 and Figure 6.

As shown in Figure 5, a minimal increase in relative risk of dengue was observed with rainfall at 50 mm per week. With the increase in rainfall form 100 mm per week to 250 mm per week, the relative risk of dengue is increasing becoming statistically significant from the lag around 8 weeks. With further increase in rainfall, the relative risk becomes significant and stronger earlier around 6 weeks. Even though, statistically not significant, the relative risk of dengue appears to be lower than 1 with more extreme rainfalls more than 350 mm per week at early lags up to around 3 weeks, while such events have a net strong positive influence on dengue at longer lags of 6–12 weeks.

As shown in Figure 6, it is clear that the relative risk of dengue sharply increased with increasing rainfall starting after 6 weeks of lag. At lower rainfall levels the relative risk becomes significant at longer lags while at higher rainfall levels it becomes significant at shorter lags and more pronounced at longer lags. The longer the lag the higher the relative risk and the highest risk was observed at around 10 weeks. At 0-week lag, the risk appeared to be decreasing compared to the reference for rainfall levels more than 300 mm per week.

Figure S2 (See supplement) shows the rainfall-dengue associations in each MOH division obtained through first stage division specific models.

### 3.2. Temperature-Dengue Association

**Figure 7:**
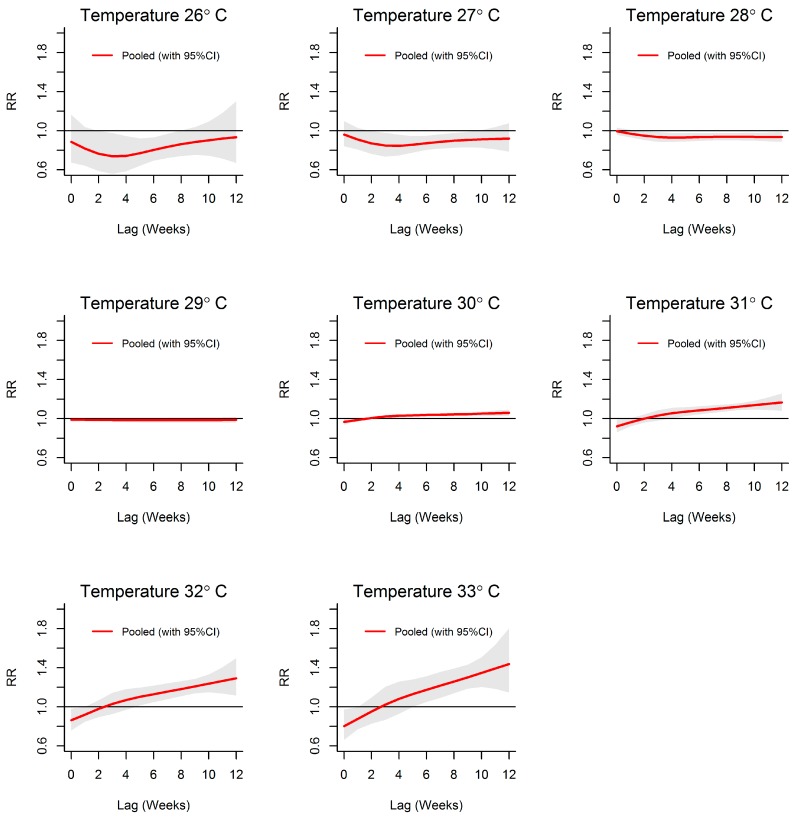
Relative risk (RR) of dengue by lag at specific weekly mean temperature levels predicted from the pooled exposure–response function for the 10 MOH divisions in Kalutara district, Sri Lanka, 2009–2013. The figure illustrates the predicted curves from meta-regression at specific temperatures (26, 27, 28, 29, 30, 31, 32, and 33 °C). Shaded areas represent 95% confidence intervals. All estimates for temperature relates to a reference of 29.8 °C.

**Figure 8:**
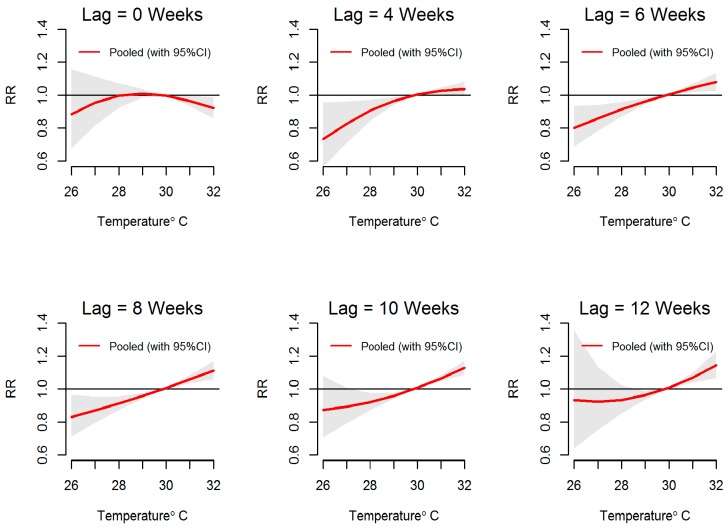
Relative risk (RR) of dengue by weekly mean temperature at specific lags in 10 MOH divisions in Kalutara district, Sri Lanka, 2009–2013. The figure illustrates the predicted curves from meta-regression for different lag periods; 0, 4, 6, 8, 10 and 12 weeks. The shaded areas represent 95% confidence intervals. Reference value is at 29.8 °C.

Figure 7 and Figure 8 show that when temperature increased, the relative risk of dengue increased over its full range in reference to the relative risk at a mean temperature of 29.8 °C. When temperature was below this reference temperature the relative risk of dengue was reduced, while above the reference value and at extreme temperatures, the relative risk of dengue was increased to 1.2 at lag 4–12 weeks. An approximately linear increase in the relative risk of dengue was observed with increasing temperature.

### 3.3. ONI-Dengue Association

**Figure 9:**
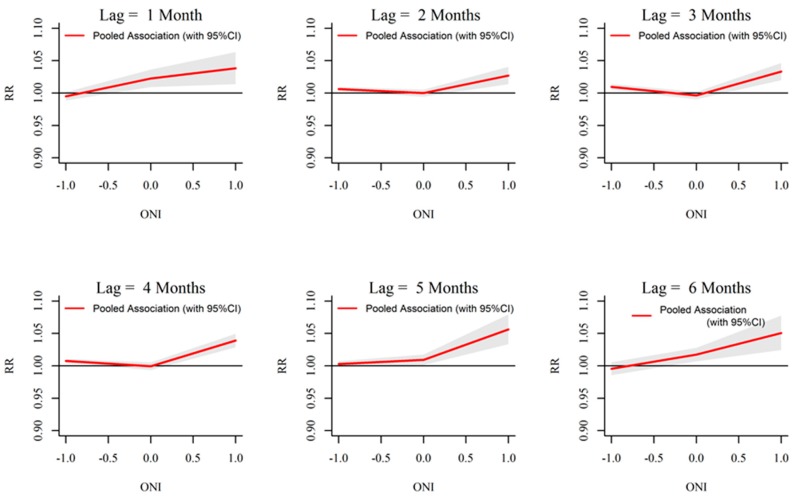
Relative risk (RR) of monthly mean temperature by ONI at specific lags in 10 MOH divisions in Kalutara district, Sri Lanka, 2009–2013. The figure illustrates the predicted curves from meta-regression for different lag periods; 1, 2, 3, 4, 5 and 6 months. The shaded areas represent 95% confidence intervals.

**Figure 10:**
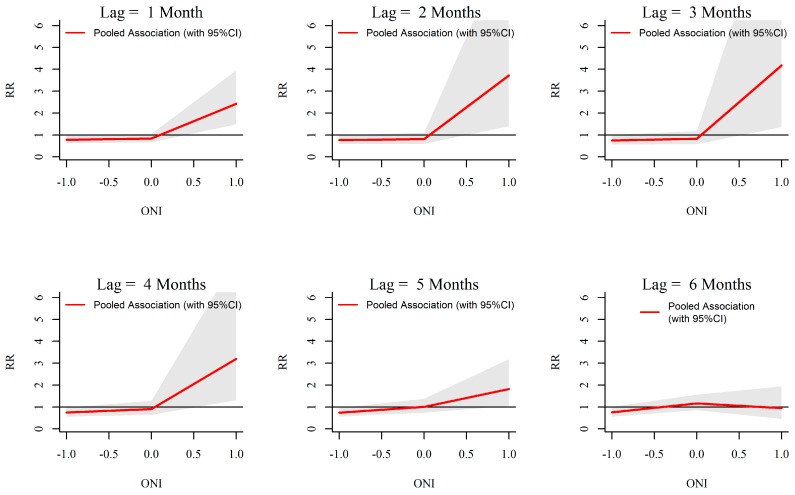
Relative risk (RR) of monthly mean rainfall by ONI at specific lags in 10 MOH divisions in Kalutara district, Sri Lanka, 2009–2013. The figure illustrates the predicted curves from meta-regression for different lag periods; 1, 2, 3, 4, 5 and 6 months. The shaded areas represent 95% confidence intervals.

**Figure 11:**
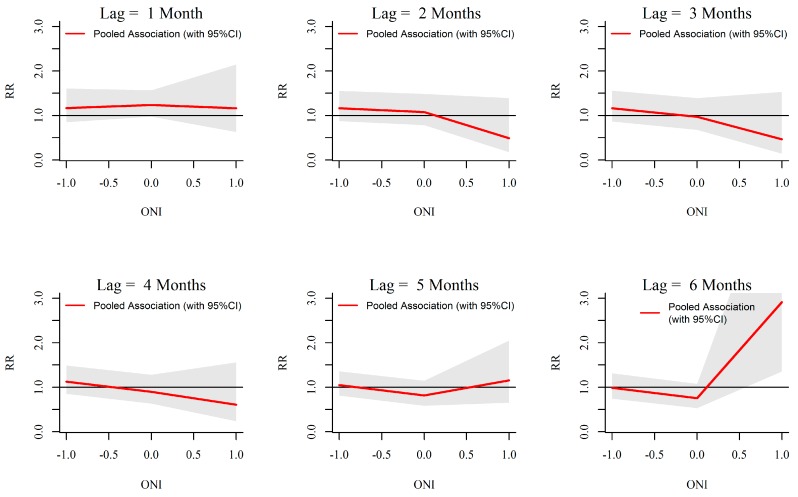
Relative risk (RR) of monthly dengue cases by ONI at specific lags in 10 MOH divisions in Kalutara district, Sri Lanka, 2009–2013. The figure illustrates the predicted curves from meta-regression for different lag periods; 1, 2, 3, 4, 5 and 6 months. The shaded areas represent 95% confidence intervals.

Analysis of rainfall and dengue association with ONI showed that extreme ENSO events (ONI more than 0.5) are significantly associated with temperature, rainfall and dengue in Kalutara. For the temperature, a significant increase in relative risk is seen starting from early as 1 month and extending up to 6 months with ONI more than 0.5 (Figure 9). Increasing ONI more than 0.5 caused increased relative risk of rainfall from a lag period of one to four months. The maximum intensity was seen around three months (Figure 10). Relative risk of dengue was seen to be significantly increasing with ONI more than 0.5 at a lag of six months (Figure 11).

### 3.4. Assessment of Heterogeneity in Associations among MOH Divisions

The overall estimated heterogeneity of the pooled exposure-response curves amounted to a Cochran Q test value of 105.7 (*I*^2^—74.5% and *p* value < 0.000) for rainfall and a value of 52.9 (*I*^2^—66% and *p* value < 0.000) for temperature indicating substantial differences between the MOH divisions in the estimated location specific responses. A closer assessment of the heterogeneity revealed that it was larger for very low and very extreme rainfall values, and for lower and average temperatures. Higher than average temperatures exerted a more consistent positive effect on dengue incidence.

Table S2 in the supplement shows that substantial heterogeneity existed among 10 MOH divisions for different exposure levels of rainfall. Lowest *I*^2^ and least significant Cochran Q test were observed for a rainfall level of 300 mm per week. Higher *I*^2^ values with highly significant Q test were seen for rainfall values deviating from 300 mm per week.

The divisional heterogeneity for different exposure levels of temperature appeared to be decreasing with increasing temperature values. Temperatures higher than the mean value had lower heterogeneity and less significant Q test compared to lower temperatures (see Table S3 in the supplement). Figure S3 (see supplement) shows the temperature-dengue associations in each MOH division obtained through first stage division specific models.

Heterogeneity for ONI associations was not statistically significant at *p* value of 0.05. Highest *I* square statics and Q test was observed for overall ONI and dengue associations (see Table S4 in the supplement).

Variable selection was done by a preliminary analysis using generalised additive model. We have considered rainfall, relative humidity, morning (minimum) and evening (maximum) temperature and ONI as explanatory variables. We found that only the rainfall, evening (maximum) temperature and ONI are significantly (at a *p* value of less than 0.05) influencing the dengue in the study area. Relative humidity was not significant. The model selection procedure regarding the natural cubic spline functions, as described in the methodology section, used a spline with 4 knots for rainfall and a single knot for temperature. As a sensitivity analysis, we compared the results using different number and positioning of knots. The resulting exposure-response curves were very similar to those showed in Figure 5 and Figure 6 for rainfall and those in Figure 7 and Figure 8 for temperature. We added seasonality to the model to explore the variability explained by underlying seasonality if exists. This addition to the final model changed the AIC only minimally (Table S1 in the Supplementary Materials). This could be due to the seasonality of dengue is mostly explained by and related to weather. Residuals sequence plots in a majority of the MOH divisions from the first stage model indicated constant location and scale between observed and predicted dengue cases while the histogram of residuals showed approximately normal distribution and independent residuals. Please see supplement for more information (Figure S1 in the Supplementary Materials).

## 4. Discussion

Our findings showed that both rainfall and temperature were significantly associated with relative risk of dengue in the district. Increasing weekly cumulative rainfall was associated with increased dengue risk starting after 6 weeks of lag. The most significant positive association was seen at lag weeks 8–10 for cumulative rainfall above 300 mm per week. Compared with a reference value of 29.8 °C, increasing weekly mean temperature over its full range was associated with an elevated risk of dengue starting after 4 weeks of lag. The study also illustrates the presence of considerable heterogeneity in temperature and rainfall effect on dengue among 10 MOH divisions.

The lag associations observed in this study is compatible with dengue vector life cycle and transmission dynamics [8,9,10,11,12,13]. A 4–12 weeks lag period is a summation of the necessary time for dengue vectors to complete development cycle from eggs, become infected with the virus, extrinsic incubation period and biting activities in the gonotrophic cycle, and the intrinsic incubation period in the host, as well as the time for the patient attending health facility to be notified as a dengue case.

We found a strong association between both rainfall and temperature in the study district to the ONI with several months latency. We further found the ONI more than 0.5 associated to dengue cases directly with the additional time lag of rainfall and temperature to dengue. This suggests ONI may be used for informing on dengue fluctuations with longer lead times. Findings of the analysis of ONI and rainfall support the existing climatological evidence on the influence of ENSO extremes on the rainfall for the Western part of the Island. Studies conducted by the department of meteorology Sri Lanka have shown that there is a trend for below normal rainfall for the southwest monsoon season (May–September) during El Nino situation, while the trend is above normal during Second Inter-monsoon (October–November) [6,7]. This phenomenon indicates shifting of rainfall for 1 to 5 months during ENSO extremes and is compatible with above findings of ONI and rainfall. This may partly explain the maximum annual rainfall and subsequent surge of dengue cases during 2010. Relative risk of dengue was seen to be significantly increasing with ONI more than 0.5 at a lag of six months. This again highlights our previous finding on rainfall and dengue association with the lag period of 8 to 10 weeks. Highest dengue peak observed during 2009 may be due to introduction of DENV-1 in to Sri Lanka and may be further augmented by ENSO activity.

The mosquito vectors have aquatic larval and pupae stages; hence water is essential to sustain their life-cycle. Rainfall proliferate breeding grounds for mosquitoes in discarded man-made receptacles scattered outside human habitats in multiple geographical locations. The availability of ample breeding sites, in turn, leads to an increase in the number of mosquitoes. Warm temperatures further augment dengue transmission dynamics by increasing survival and biting rates and decreasing the extrinsic incubation period of the virus in mosquitoes [22,54]. Increasing temperature shortens the gonotrophic cycle and reduces the EIP from 5–33 days at 25 °C to 2–15 days at 30 °C [11]. It is shown that at higher temperatures the vector needs more frequent blood meals to complete the gonotropic cycle, and more than one gonotropic cycle during the vector’s lifecycle may increase the risk of dengue transmission [54,55,56]. With these favourable environmental conditions, it is biologically plausible that dengue epidemics could follow as early as 4 weeks form the exposure. The most significant lag dimension of 6 to 10 weeks for increased relative risk for notified dengue cases identified in our model explains the increased dengue risk observed in the district following such rainfall and temperature events. Our observation of an initial reduction in dengue risk at early lags following extremely heavy rainfalls may be related to the flushing of larvae from breeding sites, disturbing their habitats and lifecycle.

The findings of the study support the existing evidence on the association between weather variables and dengue incidence [57,58,59,60]. Many studies have identified an overall increased risk of dengue for rainfall and temperature at lag weeks 9–16 [21,61]. A two months lag period for rainfall was documented in other statistical and mathematical modelling studies [22,62,63]. The vectorial capacity of *Aedes* mosquitoes is highly dependent on temperature. Subsequently the transmission of dengue virus may increase under warmer temperatures as more mosquitoes become infectious during their lifespan [12]. Statistical inferences obtained through our study are also compatible with dengue vector biology and transmission dynamics [33].

Our study has the added advantage of identifying and contrasting the heterogeneous nature of weather-dengue associations across small geographical divisions with different social and bio-ecological factors. Such heterogeneity in local settings has not been quantified in Sri Lanka to the best of our knowledge [31,64]. We observed the presence of considerable heterogeneity in dengue risk among the 10 MOH divisions for different rainfall and temperature values. The heterogeneity was specifically related to the lower and higher range of the observed rainfall distribution and the lower and medium range of the observed temperature distribution. Particularly, higher temperatures seem to be more consistently associated with higher dengue risk. The homogeneous behaviour of the exposure-response relationship across 10 MOH divisions at moderately high rainfalls around 300 mm per week and temperatures higher than 30 °C from February to April appeared to be related to subsequent increases in dengue from May to September in the district, as seen in Figure 4. It is interesting to note that heterogeneity is not statistically significant and *I* square value is 1% for overall ONI-rainfall and ONI-temperature associations. Reasons could be that ONI is signalling a more complete and consistent weather change in the district. It is also natural to see the absence of such consistent pattern for ONI-dengue associations (*I* square statistic 27.2%) as many other factors governing dengue outbreaks.

Considerable heterogeneity might have arisen from other covariates that are not explained by weather associations. Reasons explaining the heterogeneity between MOH divisions may include differences in human behaviour, population movement patterns, herd immunity, circulating dengue viral strains, and land use. Furthermore, the effectiveness of control and prevention activities depends on community support, inter-sectoral participation, and the availability of health staff in each MOH division. Panadura, Horana, Bandaragama, Matugama, Madurawala and Walallavita divisions account for 77% of the population, 57% of the land area and 80% of the total dengue cases reported in the district during the study period. Since most of the public and private institutions, including schools and factories, are located in these divisions, there is a constant movement of people within and across these divisions. Lack of proper waste management system and poorly developed urban infrastructure along with high population densities may result in an accumulation of multiple outdoor artificial breeding places for *Aedes* mosquitoes. Panadura reported about 40% of the total dengue cases in the district during the study period. In this MOH division, the increase in the relative risk of dengue appears to be started 2 weeks prior to other divisions in the district. The division is situated adjacent to the Western margin of the Colombo, which is the most highly affected district in the island. This and abundant mosquito breeding sites and high population density might have led to the distinct pattern observed with an increased relative risk of dengue at short lags for both rainfall and temperature (see Supplementary Materials Figures S2 and S3). In contrast, Ingiriya, Bulathsinhala, Agalawatta and Palindanuwara, characterized by rain forests and mountain ranges, are more rural and reported low numbers of dengue cases during the study period. The livelihood of the people in these divisions is mainly dependent on agriculture. People are clustered in villages in which basic needs are met, where one could see a picture similar to other high risk divisions. Entomological surveillance conducted in these MOH divisions revealed a mixture of natural and manmade mosquito breeding places. These division specific characteristics might have influenced the dengue epidemiology and the observed heterogeneity of weather variables across the divisions.

A limitation of the study originates from the fact that temperature data were available from only 2 stations located in the district. This limitation was partly overcome because of the presence of a more uniform spatial pattern for temperature [63]. We found spatial correlation of 85% between our observational stations. Further the temperature observed from 2009 to 2013 in the district was always within the favourable range for mosquito development and dengue transmission. Analysis using remotely sense gridded data did not change the central conclusions of the study (see Supplementary Materials Figures S4 and S5 for validation of homogenous spatial distribution of temperature across all divisions using MODIS data). The presence of asymptomatic cases and underreporting of dengue cases are inherent limitations of a dengue incidence study. Even though the Epidemiology Unit monitors notifiable diseases in each MOH division regularly, dengue cases from private health institutions and general practitioners are still poorly captured. A sero-prevalence study conducted in Colombo, Sri Lanka, in 2009 showed that for every dengue fever case notified, an additional 30 primary dengue infections occur in the community [19]. Considering cost effectiveness with rapid diagnostics, laboratory diagnosis is not widely used particularly in government hospitals. Therefore unfortunately, since data with laboratory confirmed cases are not uniformly available for this analysis at the regional and sub-district level. Available scientific evidence suggests in Sri Lanka, that clinicians’ diagnosis for dengue at time of admission had a high sensitivity of 84.7% but the specificity of 32.5% is questionable [17]. However, this type of surveillance data is still the prevailing methods observing dengue disease patterns in most endemic countries.

Given the weight of evidence on the impact of climate change on dengue, it is widely accepted that the risk of dengue infection in tropical regions, including Southeast Asia, may intensify in the future. As an example, the El Niño driven prolonged outbreak seasons in the late 1990s may be a manifestation of the impact of climate variability on the disease [65]. Therefore, further studies on the impact of climatic factors on both regional and national dengue incidence are essential to understand what actions are needed to control the disease under aggravated conditions of climate variability and change. At the same time, it is important to consider how other factors, such as urbanization and population density [29], effectiveness of dengue control measures, viral genetic configuration, immunity status of the population and population mobility [66], co-facilitate dengue epidemics in order to strengthen dengue surveillance at local and global levels and reduce its public health burden.

## 5. Conclusions

We found that specific meteorological conditions often preceded dengue incidence and epidemics across different spatial units of the study area. There was a pronounced relationship with medium and high rainfall levels occurring 6–12 weeks before incidence, and a similar pattern following high temperatures. We also showed that extreme rainfall had a negative impact on dengue risk in the first 2–3 weeks, while the net effect at 12 weeks was largely positive. We found a similar, but more delayed, association between the Oceanic Niño Index, first to temperature and rainfall patterns in Sri Lanka, and likewise to dengue. This information can potentially benefit planning and implementation of dengue control efforts in the district by development of prediction models for early warnings using the latency in the system to predict dengue incidence up to 12 weeks ahead for observation of rainfall and temperature, and for 6 months for observations of the Oceanic Niño Index anomalies. Early warning systems can raise awareness, engage communities and inform specific dengue control measures. A similar statistical approach could be implemented to explore multivariate associations between weather and dengue in other districts and beyond, particularly to respond to outbreaks that do not following expected seasonal patterns, such as during El Niño events. Further research is needed to understand the interaction between environment, migration, control efforts and dengue. Such information may shed light on the epidemiology of dengue and the drivers of dengue transmission in its full context, provide an improved knowledge base, and lead to more accurate predictions of dengue.

## Figures and Tables

**Table 1:** Descriptive statistics of the study variables per MOH division over the study period.

MOH Division	Land Area (km^2^)	Population Density (Per km^2^)	Average Annual Dengue Cases (2009 to 2013)	Average Annual Incidences (Per 100,000 Population)	Weekly Cumulative Precipitation (Range; mm)	Weekly Mean of Evening Temperature (Range; °C)
Panadura	72	3352.4	590	235.3	50.0 (0–508)	29.43 (25.50–31.55)
Matugama	268	507.2	179	131.7	63.2 (0–569)	29.43 (25.50–31.55)
Walallavita	210	279.5	126	206.3	59.6 (0–521)	29.86 (25.72–33.63)
Horana	110	1077.9	124	108.4	53.2 (0–570)	29.86 (25.72–33.63)
Bandaragama	55	2090.5	109	95.7	53.2 (0–570)	29.43 (25.50–31.55)
Ingiriya	93	639.2	99	168.7	77.3 (0–630)	29.86 (25.72–33.63)
Madurawala	135	665.4	82	92.7	52.7 (0–521)	29.43 (25.50–31.55)
Palindanuwara	270	208.0	74	129.7	69.7 (0–460)	29.86 (25.72–33.63)
Bulathsinhala	210	333.8	52	76.1	77.3 (0–630)	29.86 (25.72–33.63)
Agalawatta	88	414.8	49	121.3	52.7 (0–521)	29.86 (25.72–33.63)

## Supplementary Materials

The following are available online at www.mdpi.com/1660-4601/13/11/1087/s1. Table S1: Quasi AIC changes associated with inclusion/exclusion of weather variables, Figure S1: Residual Diagnostic Plots of the model, Table S2: Cochran Q test and *I*^2^ statistic for second stage multivariate model based on different exposure levels of weekly cumulative rainfall, Table S3: Cochran Q test and *I*^2^ statistic for second stage multivariate model based on different exposure levels of weekly mean temperature, Table S4: Cochran Q test and *I*^2^ statistic for second stage multivariate model for overall cumulative association in 10 MOH divisions for ONI for each variable, Figure S2: Division specific effect of rainfall on the relative risk of dengue in 10 MOH divisions in Kalutara district showing divisional heterogeneity, Figure S3: Division specific effect of temperature on relative risk of dengue in MOH divisions in Kalutara district, Figure S4: Relative homogeneity of daytime temperatures across MOH Divisions within Kalutara during the study period, Figure S5: Relative homogeneity of nighttime temperatures across MOH Divisions within Kalutara during the study period.
