# Supplementary material for: A Spatial Hierarchical Analysis of the Temporal Influences of the El Niño-Southern Oscillation and Weather on Dengue in Kalutara District, Sri Lanka
Source: Int J Environ Res Public Health. 2016 Nov 4;13(11):1087. doi: 10.3390/ijerph13111087 (PMC5129297; doi:10.3390/ijerph13111087)
Supplement: Supplementary file 1 [file ijerph-13-01087-s001.pdf]

Supplement Materials: A Spatial Hierarchical Analysis of the Temporal Influences of Weather on Dengue in Kalutara District, Sri Lanka

Prasad Liyanage, Hasitha Tissera, Maquins Sewe, Mikkel Quam, Ananda Amarasinghe, Paba Palihawadana, Annelies Wilder-Smith, Valéerie R. Louis, Yesim Tozan and Joacim Rocklöv

Within the dlnm framework, we calculated the quasi Akaike information criterion (Q AIC) for each MOH division as a measure for goodness of model fit. The best fitting model, with the lower Q AIC explains more variability compared to other models. The difference between the combined model, containing all variables, and each model can be taken as a relative measure of the extent to which each weather variable contribute to explain variability. We selected the Model 6 with lowest sum of Q AIC for the analysis of exposure response associations in the study. The Table S1 below shows the Q AIC difference with different variable combinations.

Table S1. Quasi AIC changes associated with inclusion/exclusion of weather variables.

Model	Variable	Sum of Q AIC	Q AIC Difference to Model 6
Model 1	Time trend only	12,121.11	1915.59
Model 2	Time trend + Morning temperature	10,988.86	783.34
Model 3	Time trend + Mean temperature	10,897.73	692.21
Model 4	Time trend + Evening temperature	10,779.98	574.46
Model 5	Time trend + rain	10,598.66	393.14
Model 6	Time trend + rain + Evening temperature	10,205.52	0
**With Seasonality Included:**			
**Model**	**Variable**	**Sum of Q AIC**	**Q AIC Difference when Seasonality Included**
Model 1	Time trend + seasonality	10,802.95	1318.16
Model 2	Time trend + Morning temperature + seasonality	10,595.06	393.8
Model 3	Time trend + Mean temperature + seasonality	10,582.59	315.14
Model 4	Time trend + Evening temperature+ seasonality	10,442.48	337.5
Model 5	Time trend + rain + seasonality	10,020.38	578.28
Model 6	Time trend + rain + Evening temperature + seasonality	9998.987	206.54

When seasonality included the observed AIC change is very small in the analytical model. This implies the weather variables mostly explaining the intra annual seasonal trend in the district.

Residual Diagnosis of Distributed Lag Nonlinear Model

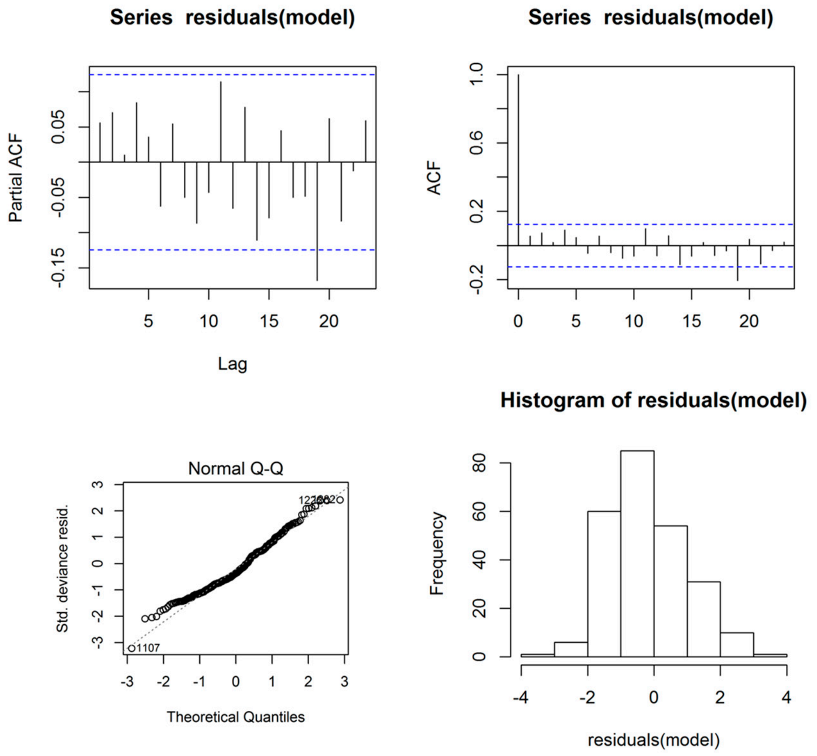

Figure S1. Residual Diagnostic Plots of the model. Upper left panel: Partial autocorrelation of the tozan@nyu.edu model. Upper right panel: Auto-correlation of the model. Bottom left panel: The normal Q-Q plot. Bottom right panel: Histogram of residuals.

Above diagnostic plots suggest reasonable model fit with the above optimal variable combination (Model 6).

Assessment of Heterogeneity in Associations among MOH Divisions

Table S2. Cochran Q test and I^2 statistic for second stage multivariate model based on different exposure levels of weekly cumulative rainfall.

Rainfall (mm Per Week)	Q Test	*p* Value	*I* Square (%)
50	89.688	0	69.9
100	96.45	0	72
150	102.994	0	73.8
200	78.614	0	65.7
250	52.492	0.0023	48.6
300	45.592	0.0141	40.8
350	49.976	0.0046	46
400	64.576	0.0001	58.2
450	75.789	0	64.4
500	78.576	0	65.6

Table S3. Cochran Q test and I^2 statistic for second stage multivariate model based on different exposure levels of weekly mean temperature.

Temperature	Q Test	*p* Value	*I* Square (%)
26 °C	67.837	0.001	46.9
27 °C	70.181	0.0006	48.7
28 °C	74.9009	0.0002	51.9
29 °C	102.901	0	65
30 °C	83.308	0	56.8
31 °C	56.262	0.0169	36
32 °C	57.005	0.0144	36.8
33 °C	58.092	0.0113	38

Table S4. Cochran Q test and I^2 statistic for second stage multivariate model for overall cumulative association in 10 MOH divisions for ONI for each variable.

Variable	Q Test	*P* Value	*I* Square (%)
Rainfall	23.15	0.9999	1.0
Temperature	31.44	0.6848	1.0
Dengue cases	74.17	0.0356	27.2

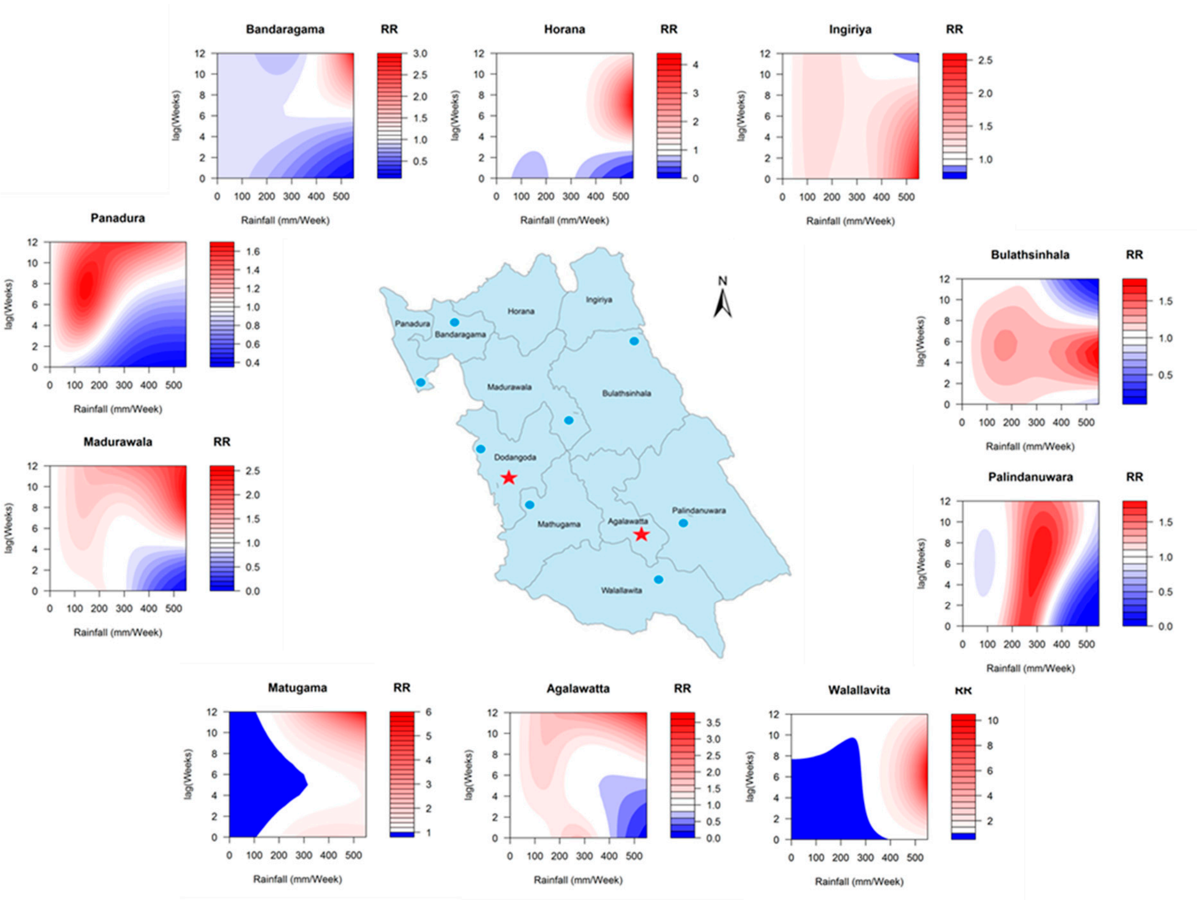

Figure S2. Division specific effect of rainfall on the relative risk of dengue in 10 MOH divisions in Kalutara district showing divisional heterogeneity. Cumulative rainfall in millimetre per week and the lag period in weeks are shown in horizontal and vertical axis respectively. The colour bar represents the intensity of the relative risk. Each contour plot shows the rainfall-dengue association in each division obtained by first stage division specific model.

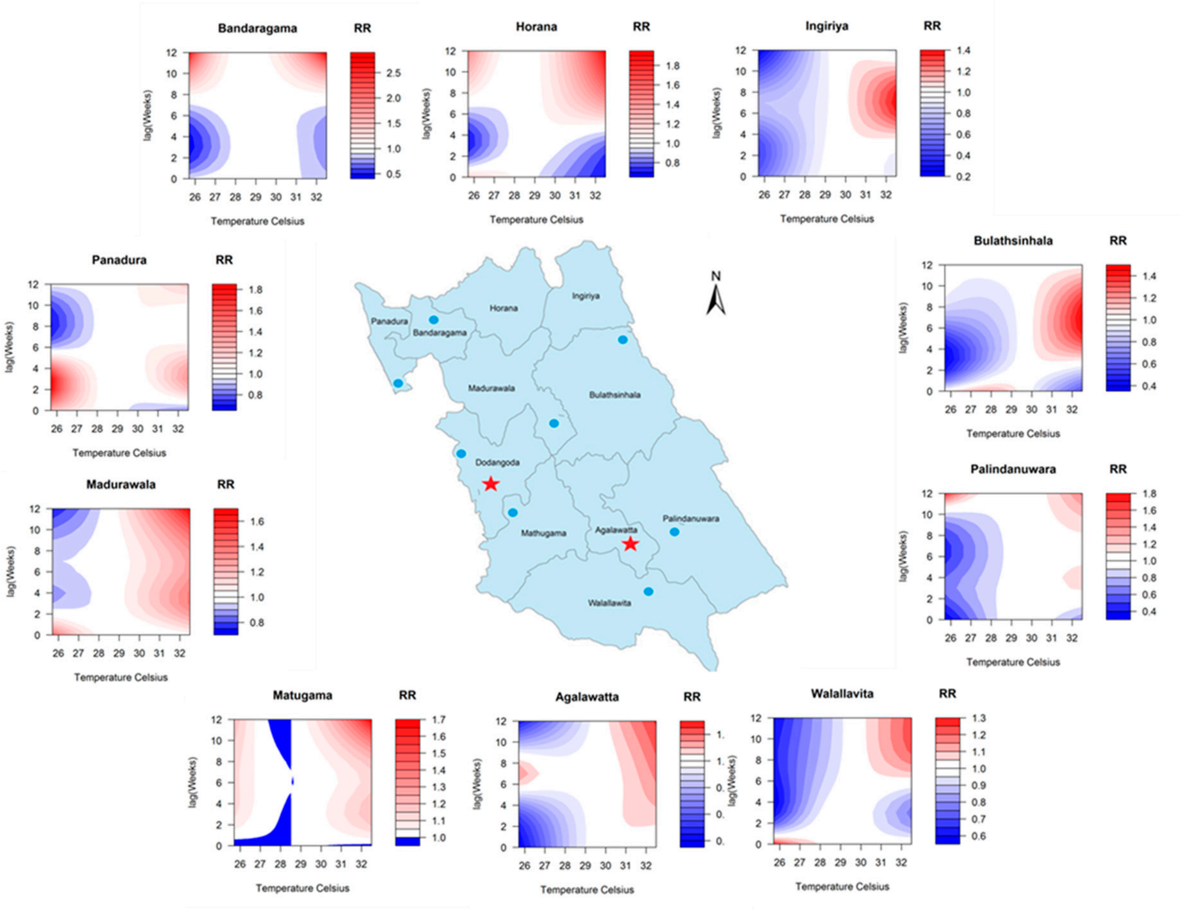

Figure S3. Division specific effect of temperature on relative risk of dengue in MOH divisions in Kalutara district. Weekly mean temperature in Celsius given in the horizontal axis. The lag period in weeks is in the vertical axis. The colour bar represents the intensity of the relative risk. Each contour plot shows the temperature-dengue association in each division obtained by first stage division specific model.

Moderate Resolution Imaging Spectroradiometer (MODIS) Land Surface Temperature and Emissivity Products Temperature Validity Verification

Since Kalutara is a small geographical area located in western costal border of Sri Lanka with 1500 square kilometers of extent (roughly about 50 km long and 30 km wide) and the altitude of populated area is within 150 m above sea level, we assumed that the distribution of temperature spatially across all divisions would be nearly homogenous.

As the figures below show, using very high resolution data for both daytime and night time, the Kalutara district (with MOH districts overlaid) is largely homogenous in terms of temperature. Here we show a very high quality data warm daytime scan (Figure S5) and nighttime scan (Figure S6) from a MODIS Land Surface Temperature and Emissivity product with 1 km * 1 km spatial resolution and approximately one week temporal resolution (8-day average, day and night, respectively.

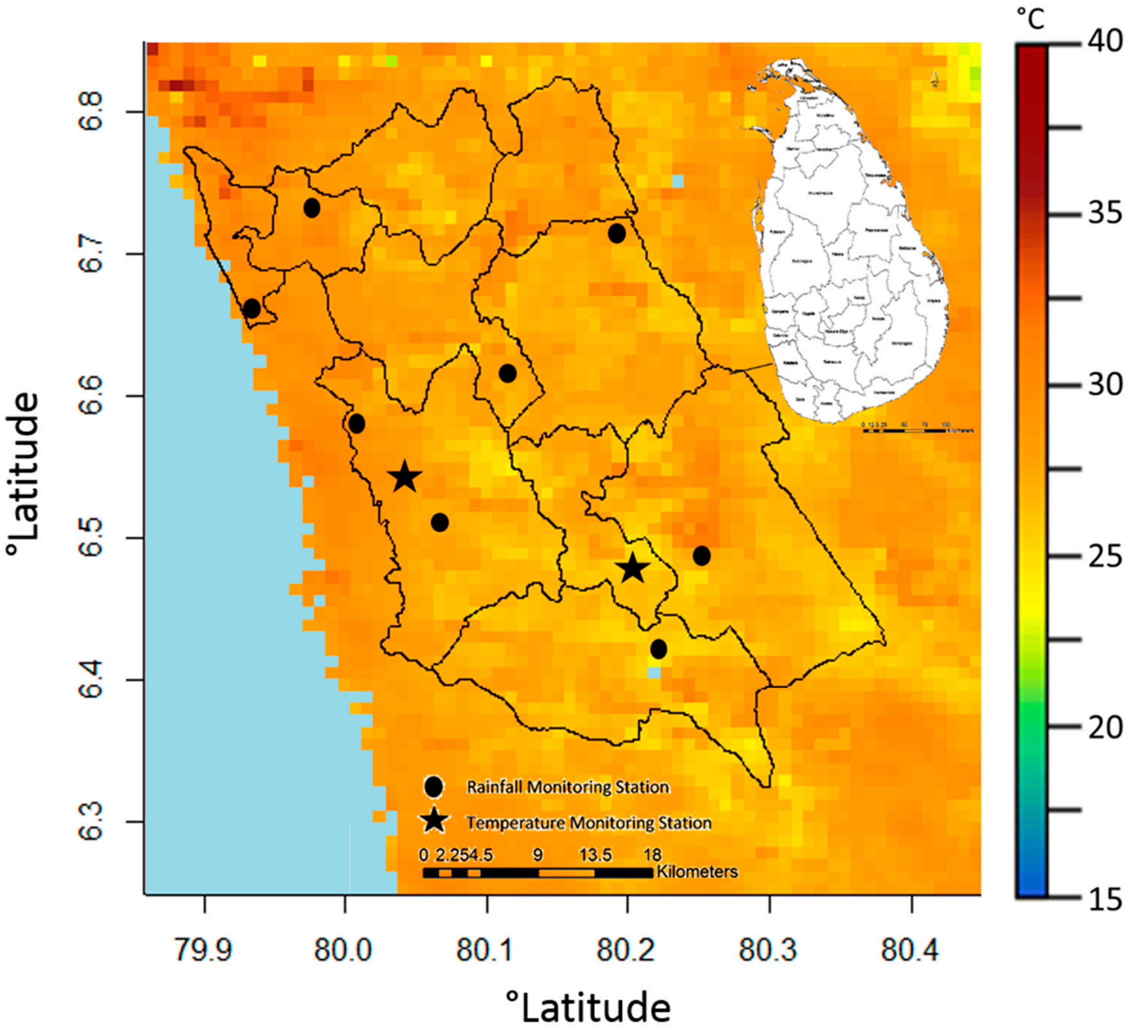

Figure S4. Relative homogeneity of daytime temperatures across MOH Divisions within Kalutara during the study period. The color scale indicates temperature the output from remotely sensed Land Surface Temperature using MOD11A2_LST_Day Scan for an 8 day period in 2013 as a demonstration of the validity of applying the relatively few temperature monitoring stations' data to perform the analysis for the multiple MOH Divisions in the study.

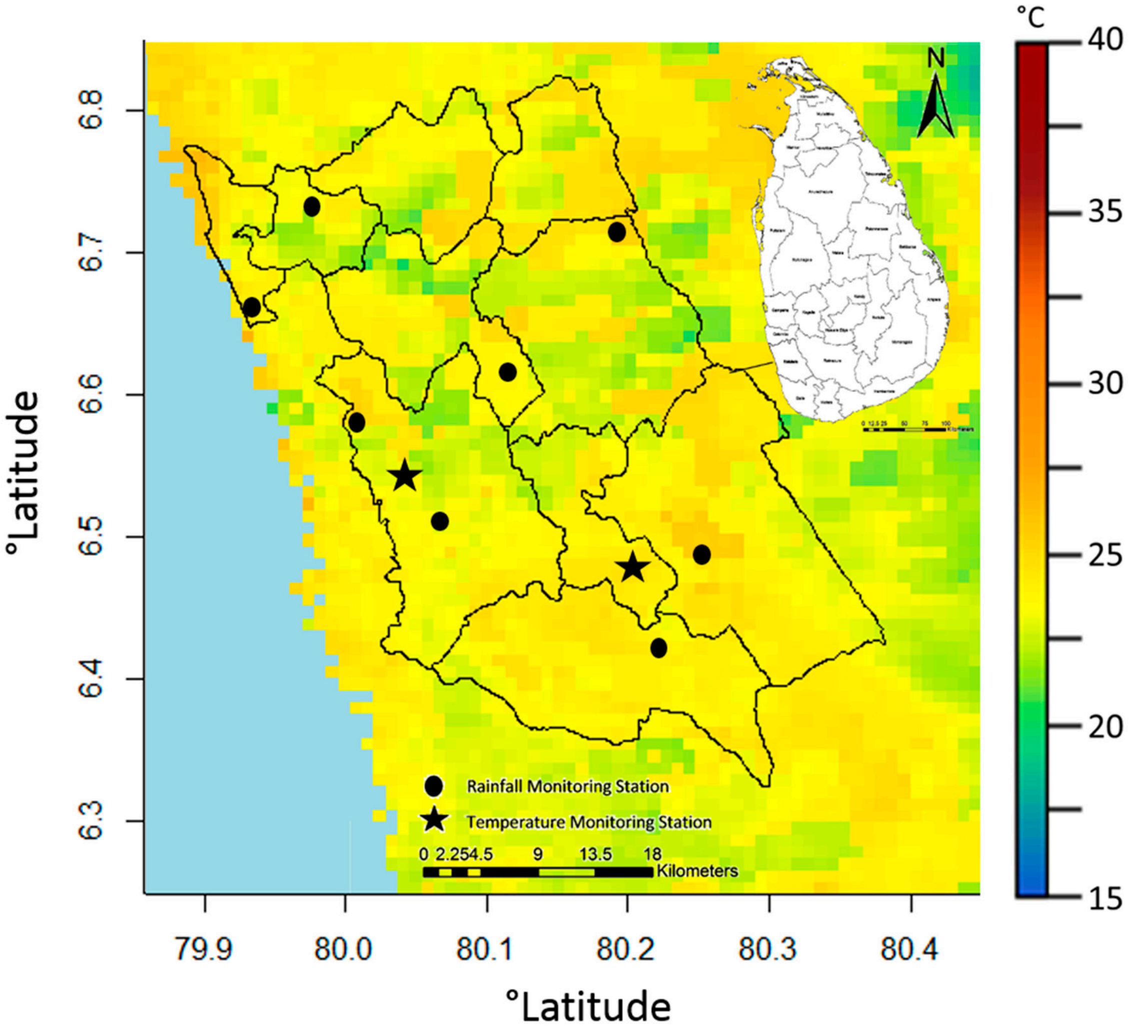

Figure S5. Relative homogeneity of nighttime temperatures across MOH Divisions within Kalutara during the study period. The color scale indicates temperature the output from remotely sensed Land Surface Temperature using MOD11A2_LST_Night Scan for an 8 day period in 2013 as a demonstration of the validity of applying the relatively few temperature monitoring stations' data to perform the analysis for the multiple MOH Divisions in the study.

© 2016 by the authors; licensee MDPI, Basel, Switzerland. This article is an open access article distributed under the terms and conditions of the Creative Commons by Attribution (CC-BY) license (http://creativecommons.org/licenses/by/4.0/).
